# Should compression bandage be performed after total knee arthroplasty? A meta-analysis of randomized controlled trials

**DOI:** 10.1186/s13018-019-1527-9

**Published:** 2020-02-14

**Authors:** Pei Liu, Xiaohong Mu, Qidong Zhang, Zhaohui Liu, Weiguo Wang, Wanshou Guo

**Affiliations:** 1 Beijing University of Chinese Medicine, Yinghuadong Road, Chaoyang District, Beijing, China; 2 Department Orthopedics 4, Beijing University of Chinese Medicine, Dongzhimen Hospital, Beijing, China; 3 Department of Orthopaedic Surgery, Beijing Key Lab Immune-Mediated Inflammatory Diseases, China-Japan Friendship Hospital, Yinghuadong Road, Chaoyang District, Beijing, China

**Keywords:** Compression bandage, Total knee arthroplasty, Meta-analysis

## Abstract

**Background:**

Compression bandage often is used after total knee arthroplasty (TKA) to alleviate pain, ameliorate swelling, and reduce bleeding. However, there is controversy about its application due to conflicting clinical outcomes and potential compression-related complications. This meta-analysis aimed to answer the question of if compression bandage should be implemented routinely after TKA.

**Methods:**

Relevant randomized controlled trials (RCTs) on compression bandage were comprehensively retrieved utilizing search engines such as PubMed, EMBASE, Web of Science, and the Cochrane Library, up to September 2019. Studies included in the meta-analysis were those that compared post-operative pain score, swelling, total blood loss, pre- and post-operative hematocrit levels differences, range of motion (ROM), and complications, using Review Manager 5.3.0.

**Results:**

Included were seven RCTs, which reported on 511 knees. The pooled results showed the compression bandage group was associated with a greater post-operative pain score during ambulation at 48 h (WMD = 0.70, 95% CI 0.07 to 1.34, *P* = 0.03), compared with the non-compression bandage group. No statistically significant differences were found between the groups in post-operative pain scores at the other times, swelling, blood loss, ROM, or other complications (*P* > 0.05).

**Conclusions:**

The current evidence is unable to conclude that compression bandage is necessary after primary TKA. Surgeons routinely undertaking compression bandage should deliberate whether there is enough clinical evidence.

## Introduction

Total knee arthroplasty (TKA) is a safe and effective procedure for the alleviation of pain, correction of deformity, and restoration of knee function to patients with symptomatic end-stage knee osteoarthritis [1]. However, post-operative pain, swelling, and inflammation in the perioperative tissues can cause many adverse events. This includes increased wound complications, impaired knee-extension strength, slower rehabilitation, prolonged hospitalization, and even the potential risk of mortality and morbidity [2–4]. Many strategies have been investigated in attempt to manage complications, which include minimally invasive procedures, without intraoperative tourniquet utilization, intra-articular injection of tranexamic acid and/or corticosteroid, cryotherapy, immobilization, compression bandage technique, and elevation of the affected limb [5–8].

The application of compression bandages from toes to mid-thigh is common following TKA [9], with authors reporting that the compression bandages limited pain by immobilization of the affected area, ameliorated soft tissue swelling by helping lower limb venous reflux, and reduced post-operative bleeding by compressing capillaries [10–12]. However, a number of studies have shown no difference in the levels of pain, swelling, blood loss, or range of motion (ROM) after surgery when comparing the use of compression bandages with non-compression bandages [13–15]. Furthermore, the use of compression bandages may even induces further detrimental effects directly linked to the compression such as discomfort, peroneal nerve paralysis, pressure ulcers, bruising, dermal blisters, and therefore additional costs [16–18].

The clinical outcomes on whether or not the use of compression bandages is advantageous in TKA recovery have been compared in previous randomized controlled trials (RCTs); however, they have been limited by small sample sizes and heterogeneous methodology. Therefore, the aim of this research was to re-evaluate the efficacy and safety of compression bandages for use in primary TKA post-operative care using meta-analysis methods.

## Methods

This meta-analysis was carried out according to the Preferred Reporting Items for Systematic Reviews and Meta-analyses (PRISMA) checklist. No ethical approval or patient written informed consent was required.

### Literature search

Articles published in English on compression bandages after primary TKA were retrieved in electronic databases. Databases used were PubMed, EMBASE, Web of Science, and the Cochrane Library, up to September 2019. Additionally, bibliographies of articles identified as relevant were examined further to determine any other potentially relevant studies. A structured search was implemented using the following search string: (Total Knee Arthroplasty OR Total Knee Replacement) AND (Compression Bandage OR Modified Robert Jones Bandage OR Compression Therapy OR Compression Dressing) AND (Randomized controlled trials OR Random OR Blind). The search did not cover restrictions regarding publication time.

### Inclusion and exclusion criteria

RCTs were selected for the meta-analysis if the following criteria were met. Criteria included population, intervention, comparison, outcome, and study design (PICOS). Population: patients had an existing diagnosis of knee osteoarthritis and were prepared for primary TKA. Intervention: compression bandage of lower limbs. Comparison: placebo or conventional wound dressing after TKA. Outcomes: post-operative pain score, swelling, total blood loss, pre- and post-operative hematocrit levels differences, ROM, and complications. Study design: RCTs.

If any of the following was present in the study (non-conformance to inclusion criteria, low-quality RCTs and non-RCTs, conference abstracts and duplicates, undefined samples or grouping, non-therapeutic clinical studies, non-original studies, case reports, and non-full-data analysis), the study was excluded.

### Data extraction

The following data was extracted from trials (author’s name, year of publication, sample size, age, gender, body mass index (BMI), intervention, control group, outcomes, study design, and follow-up duration), using a standard data extraction form. As well, relevant data was extracted independently by two authors (Xiaohong Mu and Qidong Zhang). In studies where data was incomplete, missing, or unclear, attempts were made to contact the authors for further clarification.

### Assessment of methodological quality

Two reviewers (Zhaohui Liu and Qidong Zhang) independently determined the method quality regarding bias in the selected studies (in accordance with the Cochrane Handbook for Systematic Reviews of Interventions). Methods included in the bias assessment were random sequence generation, allocation sequence concealment, blinding of participants and outcome assessors, incomplete outcome data, reporting bias, and other bias. Subsequently, each item was scored as “yes” (low risk of bias), “unclear” (unclear risk of bias), or “no” (high risk of bias). Discrepancies were then cross-checked and resolved by a third reviewer (Weiguo Wang), of which only then was a final consensus reached. A risk-of-bias summary and risk-of-bias graph was then generated using Review Manager 5.3.0 software (Nordic Cochrane Centre, Cochrane Collaboration, Copenhagen, Denmark).

### Statistical analysis

The results of eligible studies were pooled for meta-analysis when two or more results were available. Continuous variables were entered as means and standard deviations, and dichotomous outcomes as the number of events. Continuous outcomes were expressed as weighted mean differences (WMD), and dichotomous data as relative ratios (RR), and both reported to confidence intervals (CIs) of 95% (level of statistical significance *P* < 0.05). A fixed-effects model was utilized if the chi-square test showed *I*^2^ was < 50% and *P* was > 0.1, and this was used to estimate statistical heterogeneity. If these conditions were not met, a random-effects model was utilized. Unfortunately, publication bias was not assessed due to scant selected studies.

## Results

### Search results

**Fig. 1:**
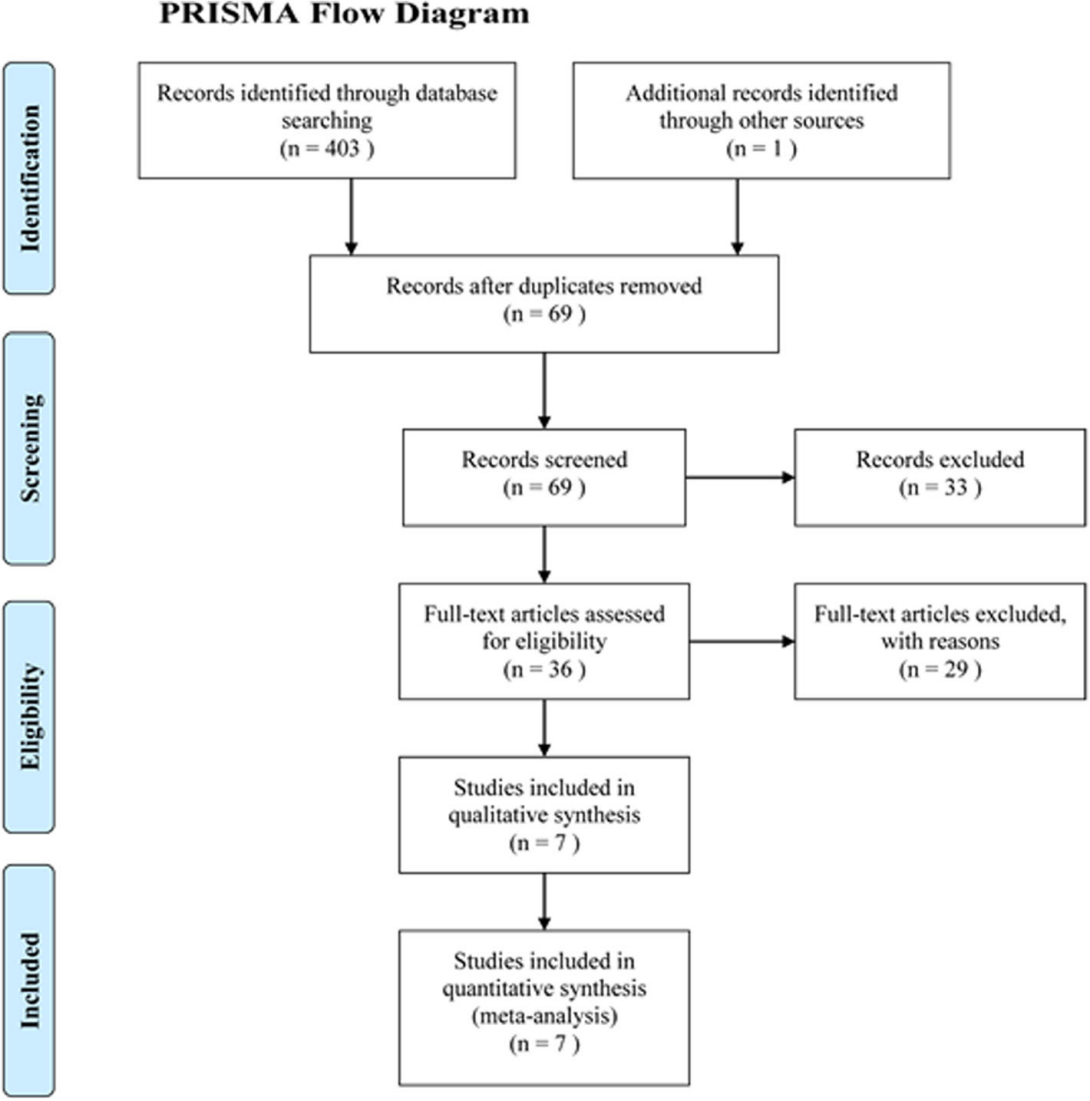
Flow diagram of study search and inclusion criteria

Initially, a total of 403 pertinent studies were identified with our search strategy, as well as an additional report found during the manual search of references. Endnote Software (Version X8, Thompson Reuters, CA, USA) detected 335 duplicate studies, thus were removed. As well, an additional 33 studies were ruled out by screening the title and abstract. Lastly, another 29 studies were excluded after reading the full text because they did not meet inclusion criteria. Therefore, only seven studies were finally selected in this meta-analysis. The PRISMA flow diagram is presented in Fig. 1.

### Description of included studies and quality assessment

A total of seven RCTs [13–15, 18–21] totaling 511 knees were involved in the meta-analysis. The average age was 67.6 years, length of follow-up term varied from 1 week to 6 months, and post-operative pain scores at rest and during ambulation were rated on the visual analogue scale (VAS, scored from 0 to 10). Post-operative swelling was quantified by circumference at the middle thigh, knee, and mid lower leg. Indicators of blood loss included total blood loss and pre- and post-operative hematocrit levels differences. Lastly, ROM and complications after surgery were reported in three and two studies respectively. Detailed information can be seen in Additional file 1.

**Fig. 2:**
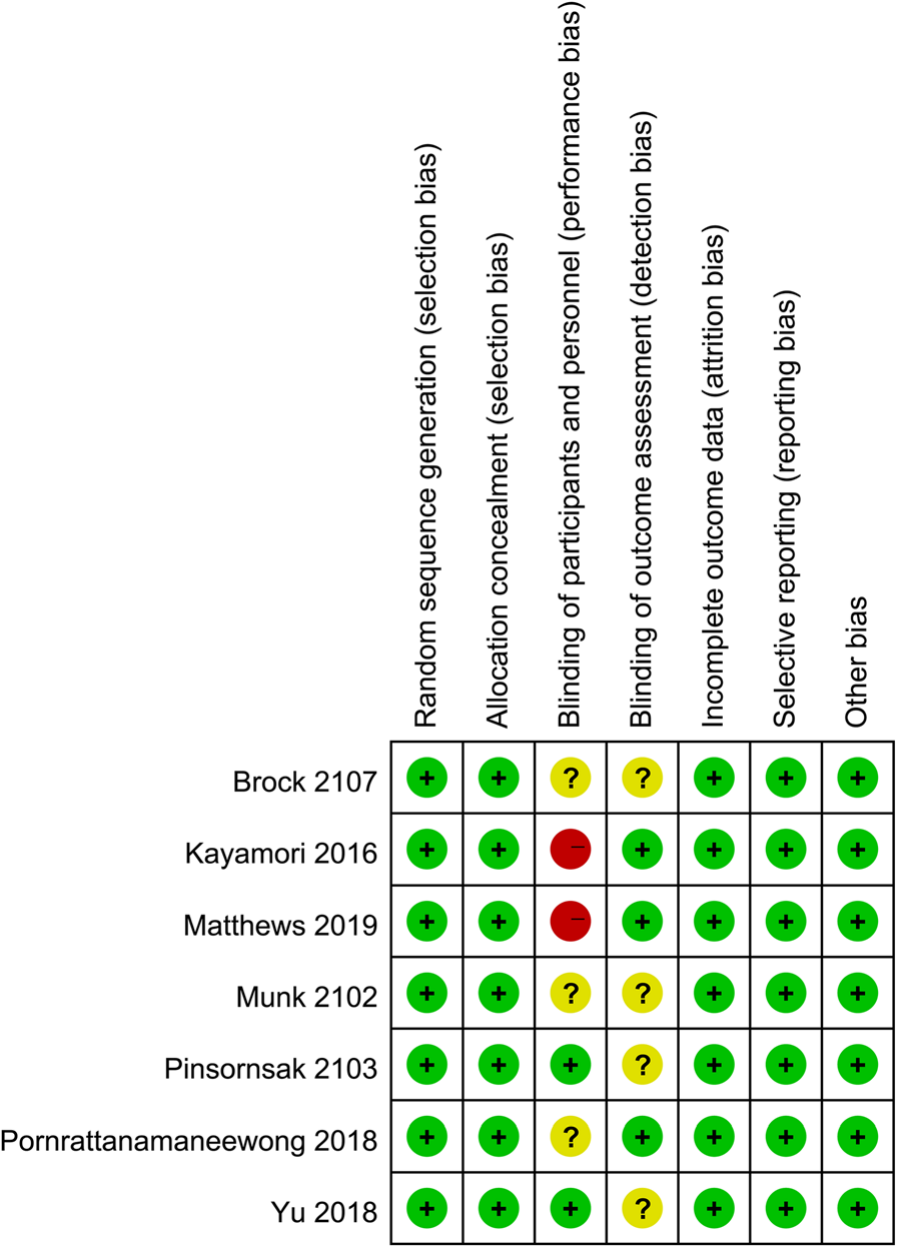
Risk-of-bias summary of included randomized controlled trials. +, no bias; −, bias,;? bias unknown

The evidence quality was evaluated using the Grading of Recommendations Assessment, Development, and Evaluation (GRADE) approach, as described in the Cochrane Handbook for Systematic Reviews of Intervention. Selection and performance bias had not been eliminated, as it was not possible to blind patients or surgeon to the intervention. However, all the selected studies contained high-quality evidence with a low risk of bias Fig. 2.

### Results of meta-analysis

#### Post-operative pain

The pain score at rest was recorded at 24 h (five studies [13–15, 19, 20] including 369 knees) and 48 h (four studies [13, 15, 19, 20] including 309 knees) post-operatively. No statistical heterogeneity was found between the compression bandage group and control group; thus a fixed-effects model was applied. Pooled results showed no significant difference in post-operative pain at 24 h (WMD = 0.33, 95% CI − 0.12 to 0.78, *P* = 0.15) or 48 h (WMD = 0.36, 95% CI − 0.15 to 0.87, *P* = 0.16).

**Fig. 3:**
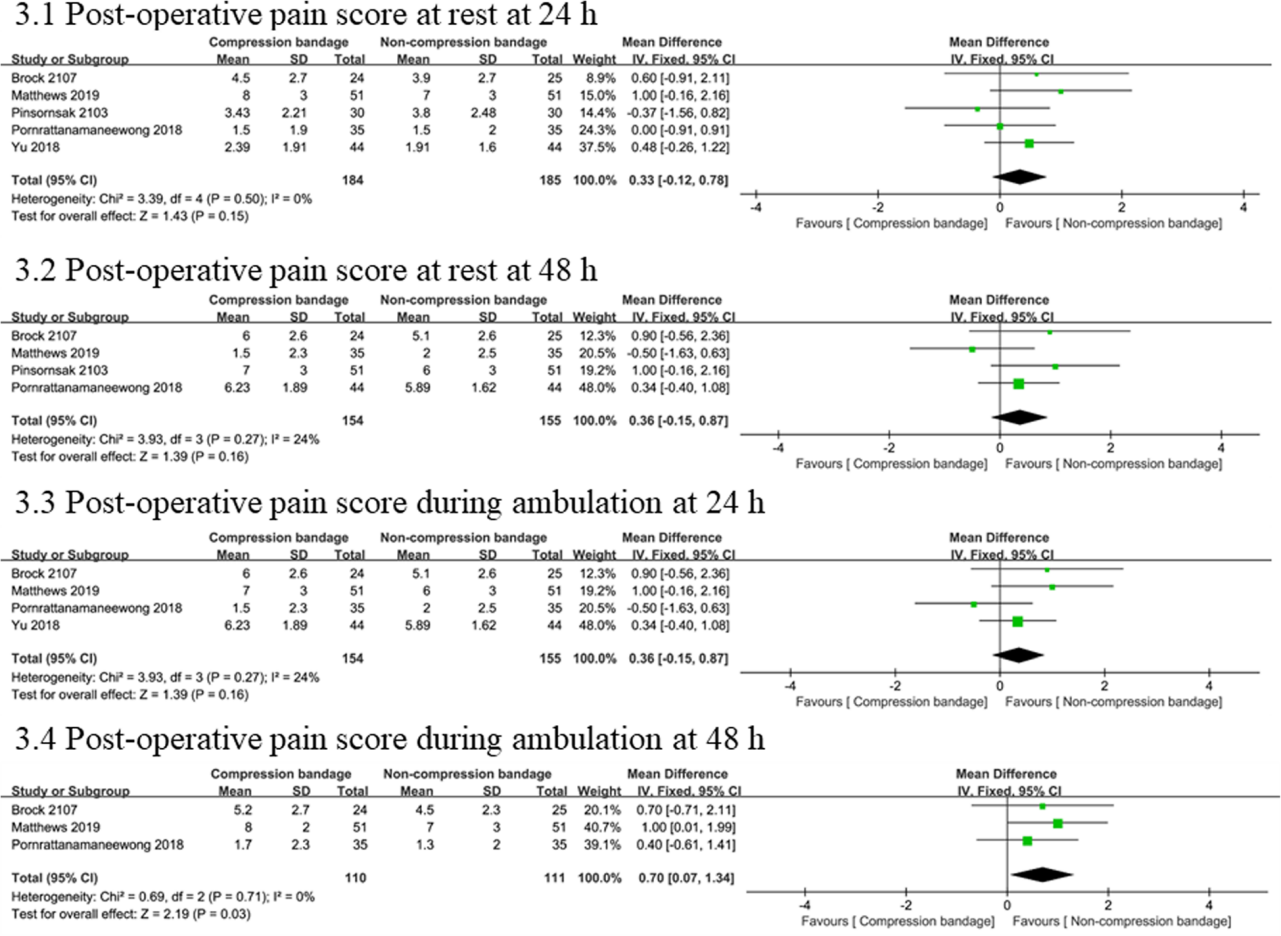
Forest plots of the included studies comparing post-operative pain

Post-operative pain during ambulation was also recorded at 24 h (four studies [14, 15, 19, 20] including 309 knees) and 48 h (three studies [15, 19, 20] including 321 knees). Similarly, tests of heterogeneity showed no difference using the fixed effects model for analysis. Pooled results showed no significant difference between both group in terms of post-operative pain at 24 h (WMD = 0.36, 95% CI − 0.15 to 0.87, *P* = 0.16). However, post-operative pain at 48 h in the non-compression bandage group was significantly lower than in the compression bandage group (WMD = 0.70, 95% CI 0.07 to 1.34, *P* = 0.03). The detailed information can be viewed in Fig. 3.

#### Post-operative swelling

**Fig. 4:**
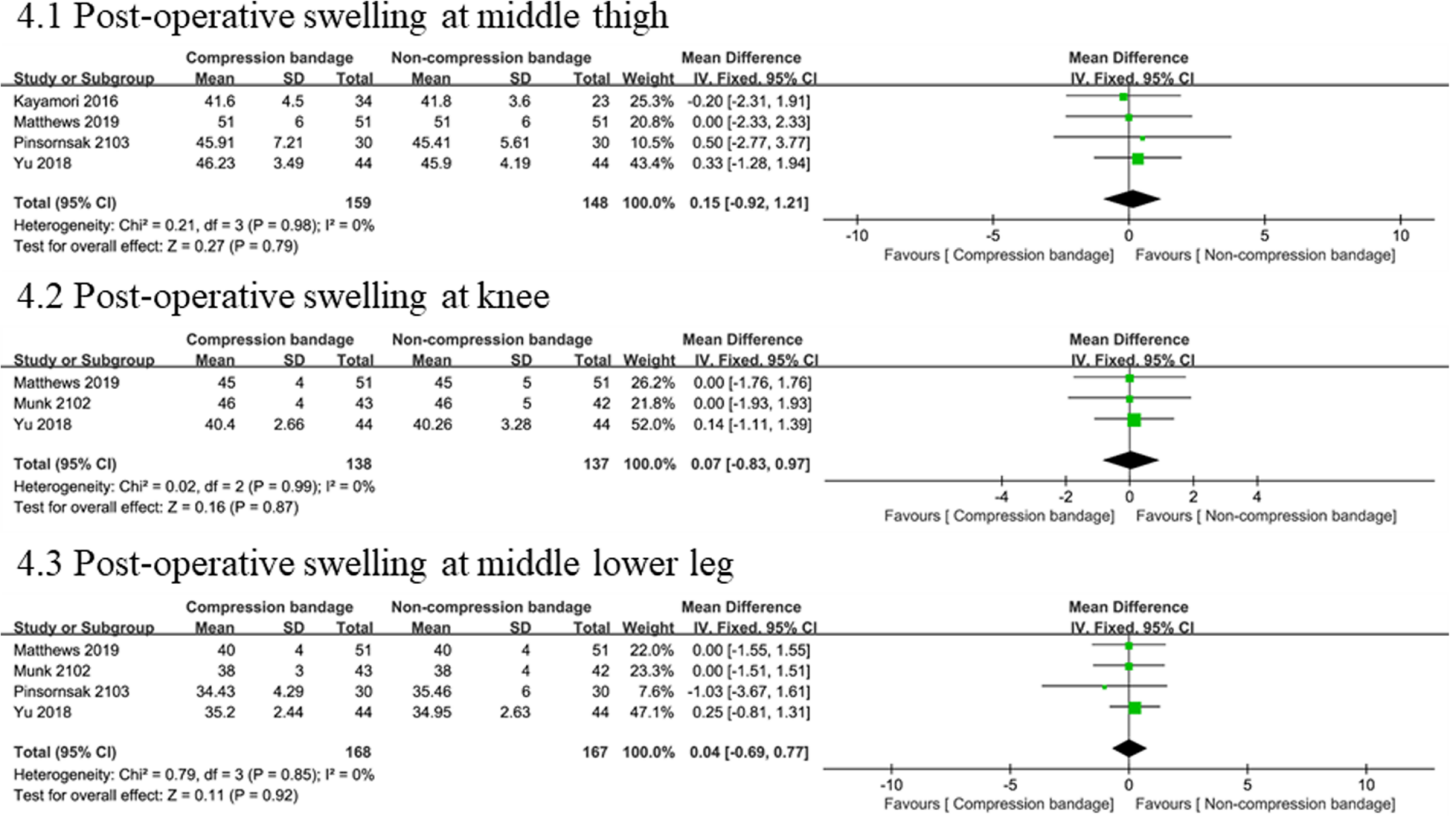
Forest plots of the included studies comparing post-operative swelling

We evaluated post-operative swelling quantified by the circumference at the middle thigh (four studies [13, 14, 19, 21] involving 307 knees), knee (three studies [14, 18, 19] involving 275 knees), and mid lower leg (four studies [13, 14, 18, 19] involving 335 knees). There was no significant heterogeneity between both groups, and thus, we used a fixed-effects model. No significant difference was found for circumferences at the middle thigh (WMD = 0.15, 95% CI − 0.92 to 1.21, *P* = 0.79), knee (WMD = 0.07, 95% CI − 0.83 to 0.97, *P* = 0.87), or mid lower leg (WMD = 0.04, 95% CI − 0.69 to 0.77, *P* = 0.92) Fig. 4.

#### Post-operative blood loss

**Fig. 5:**
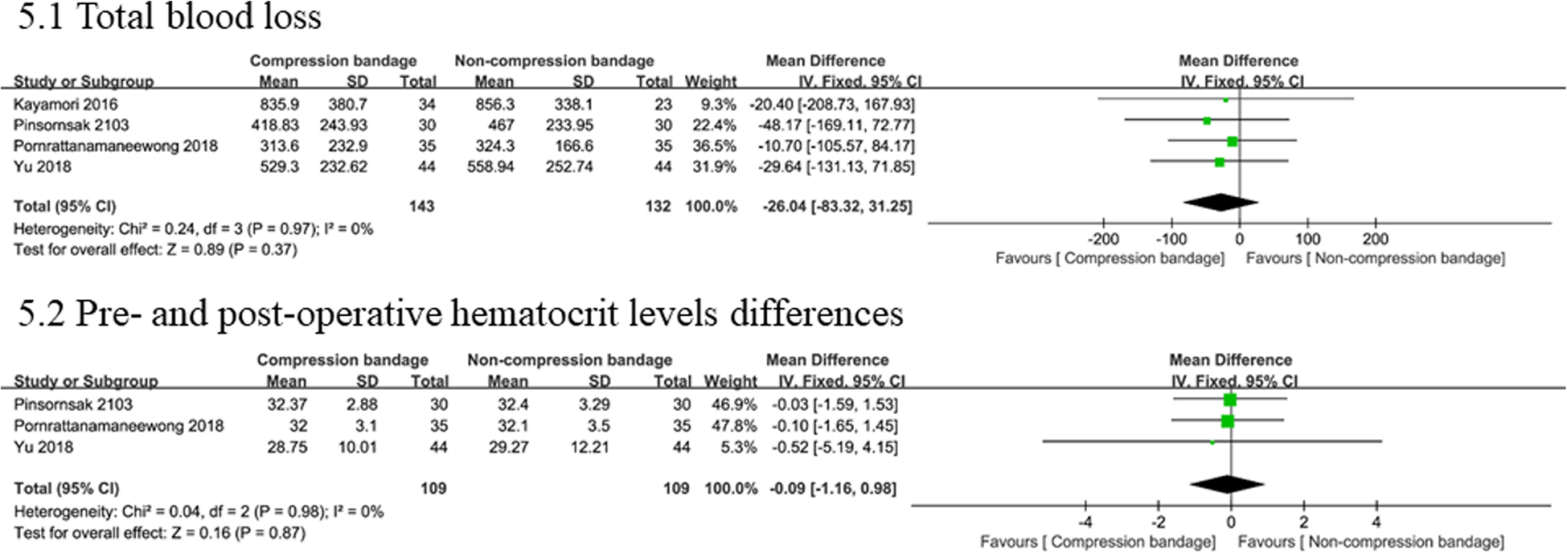
Forest plots of the included studies comparing blood loss

Post-operative blood loss was estimated via total blood loss (four studies [13, 14, 20, 21] with 275 knees), and pre- and post-operative hematocrit levels differences (three studies [13, 14, 20] with 218 knees). Pooled results indicated that the compression bandage group was not associated with less total blood loss (WMD = − 26.04, 95% CI − 83.32 to 31.25, *P* = 0.37) or pre- and post-operative hematocrit level differences (WMD = − 0.52, 95% CI − 5.19 to 4.15, *P* = 0.87). A fixed-effects model was applied according to statistical heterogeneity Fig. 5.

#### Post-operative ROM

**Fig. 6:**
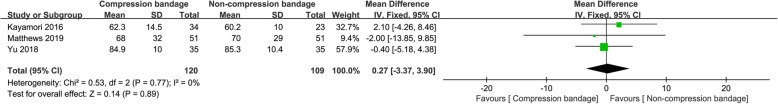
Forest plots of the included studies comparing post-operative ROM

Three of the seven RCTs [14, 19, 21] (229 knees) measured post-operative ROM in the two groups. Pooled results indicated that the use of compression bandages did not result in increasing ROM (WMD = − 26.04, 95% CI − 83.32 to 31.25, *P* = 0.37). A fixed-effects model was applied according to statistical heterogeneity (*I*^2^ = 0%, *P* = 0.77) Fig. 6.

#### Complications

**Fig. 7:**
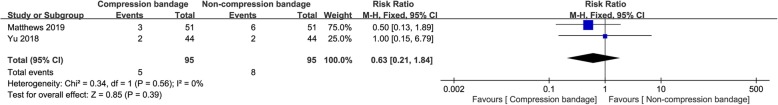
Forest plots of the included studies comparing complications

Two trials [14, 19] reported complications in a total of 190 knees. A fixed-effects model was utilized (*I*^2^ = 9%, *P* = 0.56). No significant difference in complications was observed between the two compared groups (RR = 0.63, 95% CI 0.21 to 1.84, *P* = 0.39) Fig. 7.

## Discussion

The most important findings observed in the presented meta-analysis are that compared with the use of non-compression bandage, the use of compression bandage after primary TKA generates no additional clinical benefits. Our pooled data found that the use of a non-compression bandage actually showed lower post-operative pain scores during ambulation at 48 h. It was found that there was no significant difference in regard to post-operative pain at the other times, swelling, blood loss, ROM, or complications between the two groups.

The use of compression bandages as a post-traumatic and post-operative bandage has been recommended to protect soft tissues and knee reconstruction surgery, with the therapy previously having shown positive effects in the treatments of venous ulcers and lymphedema [22, 23]. However, the potential benefits in TKA remain debatable due to conflicting results in the literature. Brodell et al. [9] showed that compression bandage could generate and sustain external compression to the soft tissues over the limb for at least 24 h, where the pressure was between 40 and 50 mmHg at initial application, decreasing to 2 to 10 mmHg within 48 h. Of which theoretically, aids venous reflux and reduces hydrostatic pressure after TKA when compared to non-compression bandage.

From previous studies, compression bandages were thought to help decrease post-operative pain [24]. Conversely, we found that post-operative pain during ambulation at 48 h in the non-compression bandages group was actually lower than in the compression bandages group (*P* = 0.03). The difference was statistically significant; however, it was not clinically relevant. Brock et al. also found a small increase in post-operative pain score in the compression bandages group at 24 and 48 h, but without a significant difference [15]. A meta-analysis including 199 knees that underwent compression bandages and 203 knees that underwent non-compression bandages concluded that there was no significant difference in post-operative pain scores [25].

Matthews et al. compared the application of an elastic compression bandage versus no bandage after TKA. They found no difference in knee swelling between the two groups, which neither benefited nor harmed the patients. Thus, they no longer use elastic compression bandage routinely for primary TKA [19]. Conversely, Pichonnaz et al. noted massive swelling after TKA within the first 2 days without using of compression bandage [26]. Our meta-analysis found no significant difference in post-operative swelling whether using the compression bandage or not. Thus, it is thought that the results of post-operative swelling may be influenced by both the method used to measure and tester. In clinical trials, swelling was usually measured by a tape, where measurement errors may occur between intra-tester and inter-tester which were reported by Jakobsen [27]. Evaluating post-operative swelling is also limited by the measurement technique. A prospective randomized study involving 19 patients with knee arthroscopy was reported by Tischer, and stated that optoelectronic measurements indicated high reliability [3].

It has been hypothesized that the use of compression bandages could have a tamponade effect and help reduce blood loss [4]. Pinsornsak et al. performed a RCT including 60 participants, and reported that the modified Robert Jones bandage tended to reduce blood loss by 46 mL compared with the conventional dressing during the first 24 h [13]. However, Gibbons et al. reported that the modified Robert Jones bandage group was associated with more blood loss than the control group using conventional dressing (1200 mL versus 720 mL, respectively) [28]. In our meta-analysis, blood loss was evaluated via total blood loss and pre- and post-operative hematocrit levels differences. Pooled results showed that the application of compression bandages did not result in a significant reduction of blood loss following TKA. Many studies agree with our findings in term of blood loss [20, 25], therefore these results combined indicate that the pressure from compression bandages is not enough to control intra-articular bleeding effectively.

Brock et al. [15] and Kayamori et al. [21] both found no differences in post-operative knee ROM in their studies. Brock reported that ROM values were close to pre-operative levels by 6 weeks after TKA [15], and a similar result was found in our meta-analysis. In contrast, Cheung et al. found improved flexion ROM and ability to straight leg raise at discharge when comparing cohesive inelastic compression bandage versus standard crepe bandage [11]. Charalambides et al. reported shorter length of stay and greater ROM on discharge when a compression bandage was used [4]. However, these studies had weaknesses in the design of retrospective data in the cohort.

Our meta-analysis demonstrates that compression bandages can be carried out safely after TKA without difference in complications between the both groups. Yu et al. found a significantly lower patient comfort level in the compression group during the first post-operative 24 h [14], and his finding agreed with Ramelet’s study, where compression therapy was related to poor patient compliance [29]. Reasons for not using compression therapy included that the bandage induced discomfort such as “cutting off” of circulation, “too hot” to wear, limb distress, poor cosmetic appearance, contact dermatitis, and itching [30].

This meta-analysis is not without limitations, and these should be acknowledged. First, only seven RCTs involving 511 patients were selected, and the limited sample sizes have weakened the objective evaluation. Second, the compression bandage techniques utilized in each RCT were not uniform. Compression bandages have different shapes, consistencies, thicknesses, and sub-bandage pressure, which may cause bias. Third, publication bias is unescapable due to the published language being identified as English. Fourth, the varied follow-up term will introduce heterogeneities, and longer-term follow-up was necessary. Finally, the included studies used different rehabilitation schemes, which could also have affected outcomes. This meta-analysis did not estimate these factors, and further evaluation is necessary in future studies.

## Conclusion

Based on this meta-analysis, we found that the outcomes between the use of a compression bandages and non-compression bandages after TKA are comparable, which neither benefit nor harm the patient. However, improvements in future meta-analysis in this area should consider larger sample sizes and high quality research to identify the validity in future.

## Supplementary information

**Additional file 1.** Raw data.

## Data Availability

The datasets generated during and/or analyzed during the current study are available from the corresponding author on reasonable request.

## Abbreviations

CI: Confidence intervals
GRADE: Grading of Recommendations Assessment, Development, and Evaluation
KOA: Knee osteoarthritis
KSS: Knee Society Score
OA: Osteoarthritis
PRISMA: Preferred Reporting Items for Systematic Reviews and Meta-analyses
RCT: Randomized controlled trials
ROM: Range of motion
RR: Relative ratios
TKA: Total knee arthroplasty
VAS: Visual analogue scale
WMD: Weighted mean differences
